# Biosynthesis of Hyaluronic acid polymer: Dissecting the role of sub structural elements of hyaluronan synthase

**DOI:** 10.1038/s41598-019-48878-8

**Published:** 2019-08-29

**Authors:** Garima Agarwal, Krishnan K. V., Shashi Bala Prasad, Anirban Bhaduri, Guhan Jayaraman

**Affiliations:** 1 Materials Simulation group, Samsung Advanced Institute of Technology, Samsung R&D Institute, Bengaluru, Karnataka 560037 India; 2 Department of Biotechnology, Bhupat and Jyoti Mehta School of Biosciences, Indian Institute of Technology Madras, Chennai, 600036 India

**Keywords:** Molecular modelling, Protein structure predictions

## Abstract

Hyaluronic acid (HA) based biomaterials have several biomedical applications. HA biosynthesis is catalysed by hyaluronan synthase (HAS). The unavailability of 3-D structure of HAS and gaps in molecular understanding of HA biosynthesis process pose challenges in rational engineering of HAS to control HA molecular weight and titer. Using *in-silico* approaches integrated with mutation studies, we define a dictionary of sub-structural elements (SSE) of the Class I *Streptococcal* HAS (SeHAS) to guide rational engineering. Our study identifies 9 SSE in HAS and elucidates their role in substrate and polymer binding and polymer biosynthesis. Molecular modelling and docking assessment indicate a single binding site for two UDP-substrates implying conformationally-driven alternating substrate specificities for this class of enzymes. This is the first report hypothesizing the involvement of sites from SSE5 in polymer binding. Mutation at these sites influence HA production, indicating a tight coupling of polymer binding and synthase functions. Mutation studies show dispensable role of Lys-139 in substrate binding and a key role of Gln-248 and Thr-283 in HA biosynthesis. Based on the functional architecture in SeHAS, we propose a plausible three-step polymer extension model from its reducing end. Together, these results open new avenues for rational engineering of Class I HAS to study and regulate its functional properties and enhanced understanding of glycosyltransferases and processive enzymes.

## Introduction

Hyaluronic acid (HA) is a hetero-biopolymer of repeating β-1,4-D-glucuronic acid and β-1,3-*N-*acetyl-D-glucosamine units^1^. It is an essential component of extracellular matrix and assists in matrix reorganization, wound repair and mediates signaling^2–6^. Its role as building block in creating new biomaterials in cell therapy, cell culture and tissue engineering is increasingly being recognized^7^. Material and biological properties of HA are determined by its molecular weight and polydispersity^8^. HA polymer is synthesized by an enzyme called Hyaluronic Acid Synthase (HAS)^9–12^.

HAS is grouped into two classes - Class I (single domain integral membrane protein) and Class II (two domain soluble/membrane anchored protein)^12,13^. Each domain in Class II enzyme is responsible for catalysis of a type of glycosidic linkage^11,14,15^. While Class II enzyme is reported only in *Pasturella multocida*, Class I enzymes are ubiquitous and more complex^11,12,16^. They are grouped under the largest glycosyltransferase family, GT-2 in CAZy database^17^ containing members of diverse substrate specificities. The family members are expected to share same overall GT-A fold and inversion mechanism to create β-linked polymer from α-linked precursors^17^. The topology of Streptococcal HAS (Class I) is determined through experiments and is found to contain six membrane regions of which four are integral and two amphipathic and a cytoplasmic domain^16^. The integral membrane regions form a pore to translocate HA. The cytosolic component is a single functional glycosyltransferase domain that is expected to hold at least six activities^12^. These include two types of UDP-substrate binding (UDP-N-acetylglucosamine, UDP-D-glucuronic acid), two HA-sugar binding (HA-glucuronic acid and HA-N-acetylglucosamine) and two transferase (transfer of glucuronic acid, N-acetylglucosamine to the growing polymer chain) activities^12^.

In order to understand the complex functioning of these inter-related activities, Weigel has proposed a pendulum model for polymerization of UDP-sugars. The UDP-sugars would get added one at a time or two at a time to the reducing end of the polymer. Depending on the number of binding sites for polymer and UDP-sugars, either one of a sequential, simultaneous or alternating mechanism would enable polymer biosynthesis^12^. The number and location of binding sites is not well characterized due to the absence of 3-D structural information. Hence molecular details on how the enzyme binds to the precursor sugars and forms HA polymer are unclear^12^.

Amino acid changes in HAS sequence have shown to influence HA polymer size/molecular weight, as well as its titer, thus making HAS a good candidate for protein engineering. Mutations can thus be used to regulate HA properties which determine their biological applications^8^. Random and directed mutation studies have been conducted in *Streptococcal* and mammalian HAS systems to identify property-influencing sites. These include point mutations in membrane region^18^, cysteine residues^19^, select conserved residues^20^, upstream and downstream regions of conserved residues^21^, and C-terminal end of the enzyme^22,23^.

The present study focuses on Class I HAS. It is a difficult system to study experimentally due to the presence of transmembrane and cytosol components, both of which are integral to its function. The three-dimensional structure of Class I HAS and active site/binding site features are not yet known. A few functional residues have been characterized through site-directed mutagenesis and chemical studies^12,20^. Often their exact role in function remains undefined. In other cases, residues are found to be conserved across diverse glycosyltransferases^24,25^. Such an assessment misses out on HAS-specific functional elements. In addition, Class I HAS has several functional differences with respect to other glycosyltransferases. HAS enzyme possesses dual substrate specificities in the same functional (glycosyltransferase) domain. Other family members synthesize a homopolymer and therefore possess single UDP-substrate specificity. Polymer elongation occurs at its reducing end in Class I HAS members^12,25^. In contrast, other characterized glycosyltransferases including cellulose synthase polymerize at the non-reducing end^24^. The implications of these differences on 3-D structure, active site architecture, substrate binding sites and mechanism is completely unknown. The objective of this study is to characterize the functional components of Class I HAS to enhance molecular understanding of polymer biosynthesis and provide a framework for rational engineering of HAS. We considered *Streptococcal* HAS (SeHAS), a biochemically characterized HAS as the representative member for analysis^9^.

Our study, for the first time, presents a three-dimensional atomic scale model of Class I HAS enzyme to gain insights on functional features. We discuss the 3-D structural features of SeHAS model. We compare Class I HAS sequences and identify 9 HAS-specific sub-structural elements (SSE) and elucidate their role in polymer biosynthesis. Docking studies with UDP-substrates in SeHAS show highly overlapping single binding sites for the two UDP-substrates. *In-silico* and mutation studies identify functional elements in SSE5 implicated in polymer binding and influencing HA production. Our studies indicate a substrate binding role for Lys-139, and a critical role for Gln-248 and Thr-283. ANM-based model is analysed to assess collective global dynamics in SeHAS. Based on ligand binding landscape and architecture of functional elements, we propose a plausible three-step molecular mechanism to extend HA polymer from its reducing end.

## Results and Discussion

### 3-D Structural features of SeHAS

A structural model of SeHAS sequence was generated using RaptorX webserver^26^. The model was obtained with cellulose synthase template (PDB: 4P00).The template enzyme shares a high functional similarity and a low sequence similarity (~15%) with SeHAS^25^. Despite the poor sequence similarity to the template, the obtained model is of high quality as indicated by the global (P value: 10^−8^) and absolute (GDT score: 52) quality measures^26^. Stereo-chemical quality assessment of the structural model indicates that 98.5% of residues reside in allowed/partially allowed region of the Ramachandran plot.

**Figure 1:**
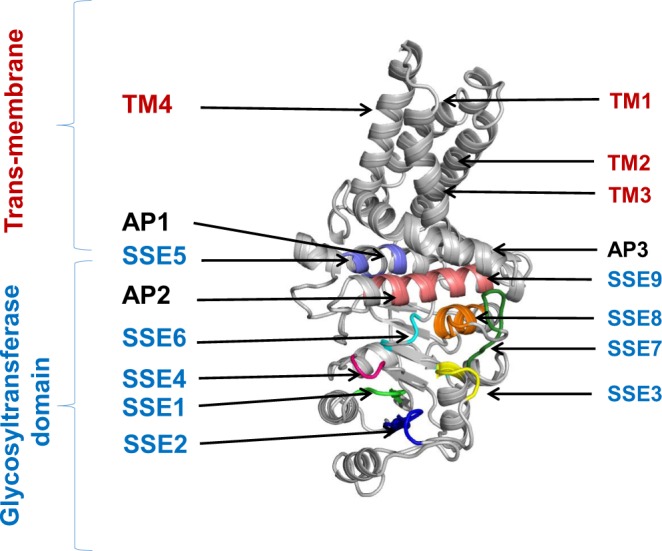
Structural model and features of SeHAS. SSE: Substructural element, AP: Amphipathic helix, TM: Trans-membrane helix.

Figure 1 shows the structural model of SeHAS. The HAS structural model shows that the single chain folds into three components: (i) a functional glycosyltransferase domain, (ii) four transmembrane helices (TM1-TM4) and (iii) three amphipathic helices (AP1-AP3). The glycosyltransferase domain adopts GT-A fold formed by mixed 7-stranded β sheet surrounded by α helices in β/α architecture. Transmembrane helices (TM1-TM4) form a four helix bundle and create a pore for polymer translocation. 11 C-terminal residues of SeHAS are predicted to be disordered and not shown in Fig. 1. The oligomeric nature of SeHAS is still being investigated. Until 2018, SeHAS was characterized to function in its monomeric form^9^. Recent reports indicate SeHAS enzyme functions as a homodimer^27^. The scope of the present work is limited to the assessment of features in the protomer.

The 3-D structural model of SeHAS is consistent with previously determined topology of HAS in *Streptococcus pyogenes* derived using fusion, labeling and protease accessibility experiments^12,16,19^ with a slight difference in the number of amphipathic helices. We propose the presence of three amphipathic helices instead of two amphipathic helices previously reported based on comparative assessment with cellulose synthase structure. SeHAS residues corresponding to amphipathic helix AP2 have not been probed specifically for their cellular location^16^. As discussed later in this manuscript, this helix holds residues of functional importance.

The structural model explains the results of N-ethylmaleimide (NEM) inhibition in SeHAS^19^. NEM reacts with thiol groups and is therefore used to probe the role of cysteine residues in proteins. SeHAS has four Cys residues: Cys-226, Cys-262 and Cys-281 and Cys-367. The first three cysteine residues are located in cytosol and are therefore accessible to NEM leading to inhibition. Cys-367, however, is located in transmembrane helix and is inaccessible to NEM. Therefore, no inhibition is reported for this residue. In the presence of substrate, NEM is shown to bind only to Cys-281. Our structural studies (described later) indicate that Cys-226 and Cys-262 are located in close proximity to UDP-substrate binding site and the presence of substrate would thus block the access of NEM to these residues. Cys-281 lies away from the UDP-binding site and the presence of substrate does not block this site from NEM inhibition.

### Substructural elements (SSE) in Class I HAS

**Figure 2:**
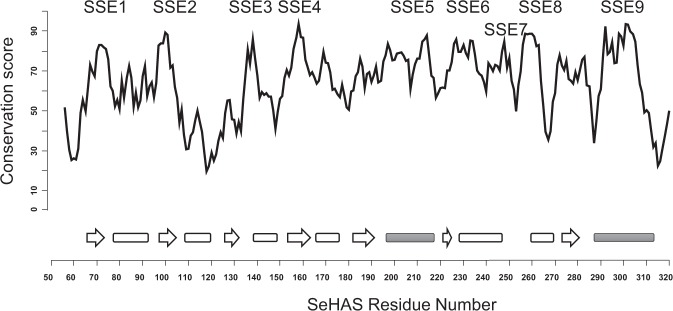
A plot of conservation score with respect to SeHAS sequence number. SSE indicated on top. The secondary structure projection of sequence is indicated at the bottom. The strands are marked as arrows and helices represented as rectangles. Filled rectangles correspond to amphipathic helices. Refer text for details.

There is little information on the functional machinery specific to Class I HAS. In the absence of 3-D structure, a functional role of experimentally studied sites is not known. Hence, to gain an understanding on the functional components in SeHAS and their relative disposition in 3-D, we identify short contiguous regions with evolutionarily conserved sequence features called sub-structural elements (SSE). We compared 81 Class I HAS sequences obtained through stringent selection criteria elaborated in *Methods* section. Class I HAS enzymes differ in the number of transmembrane helices. In the absence of structural information, the alignment in this region is of poor quality. Hence, the comparison of sequences is limited to the cytosolic region to avoid sites with ambiguous residue-residue correspondence. The alignment of HAS Class I sequences is provided as Supplementary Fig. S1, created with Jalview. A conservation score is computed for every residue-residue correspondence with respect to SeHAS sequence. A contiguous stretch with average score >=70 and at least 2 sites scoring >=80 is defined as sub-structural element (SSE). Using this criteria, we identify 9 sub-structural elements, labelled SSE1-SSE9, of potential structural/functional importance in HAS (Fig. 2). Secondary structure for most SSEs maps to loops except SSE5, SSE8 and SSE9 which occur in helices. These elements are also marked in Fig. 1. Table 1 summarizes the sequence details as well as the structural and functional role of SSE.

**Table 1:** Role of SSE in HAS class I enzymes.

S. No.	SSE No.	SSE sequence*	ResidueRange (SeHAS)	Predominant Role
1	SSE1	**Y**N**E**	74–76	Nucleotide (UDP) binding
2	SSE2	Vx**D**xS	101–105	Nucleotide (UDP) binding
3	SSE3	**GK** **R**	138–140	Substrate binding, Charge neutralization
4	SSE4	**DSD**	159–161	Metal and substrate binding
5	SSE5	**RY**xxx**F**xxx**R**	205–214	Polymer binding
6	SSE6	**CSGP**xxx**YR**	226–234	Binding site scaffold
7	SSE7	x(7)**Q**x(3) **G**	241–252	Conformation transition and stabilization
8	SSE8	**GDDR**xx**T**	258–264	Catalytic base, Stabilization of intermediate, Polymer interaction, Substrate interaction
9	SSE9	**Q**xx**RW**x**KS**xx**RE**	295–306	Substrate and Polymer binding

Our study reports conserved SSEs: SSE5 and SSE7 specific to HAS. Mutations studied so far, corresponding to residues Tyr-74, Asp-159, Ser-218, Cys-226. Leu-230, Tyr-233, Arg-234, Asp-259, Asp-260, Arg-261, Leu-263, Cys-281, Gln-295, Gln-296, Asn-297, Arg-298, Trp-299, Arg-406, Arg-413, have shown to influence activity. Mutations corresponding to residues Lys-48, Glu-327, Lys-414, Lys-415 influence reaction rate and/or molecular weight of the HA polymer^18–21,23^. Most of these mutation sites map to SSE1-4, 6, 8 and 9 (Table 1).Other sites are not a conserved feature of Class I HAS family. Bi *et al*. (2015) analysed diverse processive glycosyltransferases and discuss the presence of three variably spaced Asp residues in glycosyltransferase domain, which are crucial for activity^25^. These include, Asp from SSE1 in nucleotide binding, Asp of SSE2 in binding to metal, Asp in SSE8 with a probable role as base. In addition, a probable role in polymer binding is suggested for residues of SSE9. Weigel (2015) proposed eight Asp/Glu containing tri-peptides as potential regions involved in UDP-sugar binding^12^. In order to elucidate the role of SSE and identify regions binding to UDP-sugars, we conducted docking simulations.

HAS enzyme is expected to have at least two binding sites, one for UDP-sugar substrates and the other for polymeric sugar to catalyze a glycosidic bond formation^12^. To identify the binding sites in the enzyme structure, we conducted docking studies of UDP-sugar substrates: UDP-N-acetylglucosamine and UDP-glucuronic acid. The binding region for UDP-substrates is assessed through a grid with its center in the glycosyltransferase domain as defined in *Methods*.

### Binding sites of UDP-substrates overlap

**Figure 3:**
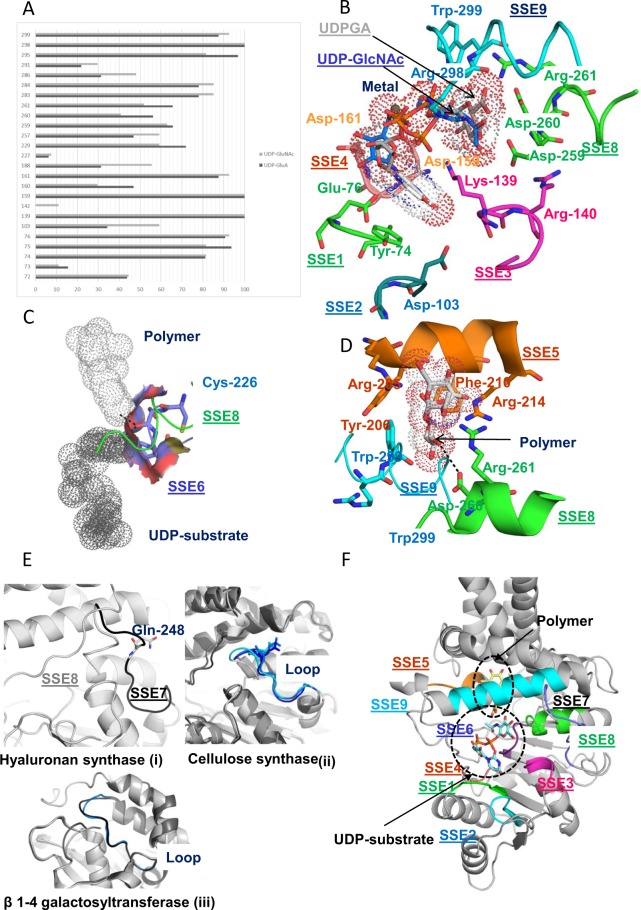
(**A**) Frequency of ligand contacting residues for energetically favourable conformers of UDP-N Acetylglucosamine (UDP-GlcNAc) and UDP- D Glucuronic acid (UDP-GlcA). Frequency is plotted on the X-axis and Sites on Y-axis. (**B**) Role of SSE1–4, SSE8 and SSE9 in UDP-sugar substrate binding. (**C**) Role of SSE6 in ligand binding. (**D**) Role of SSEs in polymer binding. (**E**) Role of SSE7. (i) SSE7 loop in hyaluronan synthase, (ii) Equivalent loop in cellulose synthase, (iii) Equivalent loop in non-processive glycosyltransferase. (**F**) Energy minimized HAS structure showing UDP-N-Acetylglucosamine and disaccharide of glucuronic acid and N-Acetylglucosamine moeities.

Docking simulations are conducted separately for UDP-N-acetylglucosamine and UDP-glucuronic acid. Low energy conformers (better than 5 kcal/mol) are selected. Figure 3A shows the frequency of contacts of various sites with energetically favourable conformers across different simulation runs. Polar residues with at least 10% frequency to any of the two substrates are shown. The notable observation from the figure is that the binding sites for the two sugar substrates overlap.

The role of the contacting residues was assessed by investigating the binding with energetically most favourable biologically relevant conformer. The selection of the conformer is guided by the proximity of nucleotide ring of UDP to the evolutionary conserved SSE1 known to be involved in nucleotide binding. The selected conformers in the binding pocket are shown in Fig. 3B. SSE1-SSE4, SSE6, SSE8 and SSE9 are involved in substrate binding (Fig. 3B). SSE1, SSE2 and SSE4 largely stabilize the uridine part of the substrates while SSE3, SSE8 and SSE9 interact with the sugar ring. Initial binding of the UDP-ring of the substrate with enzyme could be facilitated by (i) side chain of conserved Asp-103 of SSE2 (blue, Fig. 3B) through hydrogen bonds with polar groups of nucleotide and (ii) by aromatic ring of Tyr-74 (green, Fig. 3B) from SSE1 through π-π interactions with the uridine ring of UDP. Side chain of Glu-76 (green, Fig. 3B) from SSE1 forms hydrogen bonds with polar groups of ribose sugar of UDP. Residue Asp-161 from SSE4 stabilizes the OH group of ribose ring, while Asp-159 stabilizes the metal ion. Gln-295 and Arg-298 (blue) from SSE9 could interact and stabilize the pyrophosphate group of the UDP nucleotide. NE atom of Trp-299 is in H-bonding distance to the sugar substrate and likely helps in orienting the substrate. Asp-260 is topologically equivalent to the catalytic base in cellulose synthase^25^. SSE8 holds Asp-260. Sequentially proximal to this residue are Asp-259 and Arg-261 both of which interact with the polar groups of the sugar ring (green). Residue Arg-140, next to Lys-139, is partially conserved and replaced only with Lys in SeHAS homologues. This residue is in close proximity to N-terminal end of helix holding SSE8 (green). This end contains negatively charged Asp residues which could be neutralized by Arg-140.

Residues from SSE6 (Fig. 3C) create a scaffold to support ligands at the binding site. The loop is strategically placed and forms base of binding pocket. The loop also sits close to catalytic base (SSE8) and the bound polymeric sugar (discussed later). As discussed above, experiments using N-ethlymaleimide place the conserved Cys-226 of this element near UDP-sugar binding site^19^. This is likely involved in maintaining the pKa of the microenvironment at the active site. Although not essential for activity, mutation of this residue to Ala is shown to influence activity in SeHAS^19,28^. Side chain of Ser-227 is likely involved in stabilizing the loop conformation through interactions with the main chain. Gly-228 and Pro-229 provide the necessary flexibility and rigidity to the main chain conformation, respectively (Fig. 3C).

### Lys-139 is dispensable in SeHAS and has a role in substrate binding

**Figure 4:**
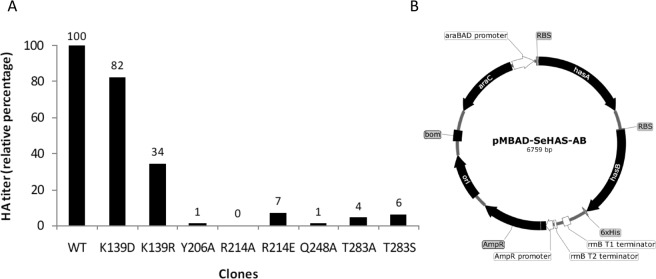
Mutation studies on SeHAS. (**A**) Relative activity of SeHAS mutants conducted in this study. Experiments were conducted in triplicates and the standard error for HA titer was in the range of ±0.01 to ±0.05. (**B**) pMBAD vector construct. *hasA* and *hasB* genes from *Streptococcus equi* subsp. *zooepidemicus* were sequentially cloned. For mutational studies, *hasA* wildtype was replaced with corresponding mutant(s).

Docking assessments suggest a role of Lys-139 in ligand binding. Previous studies have reported a regulatory role for this residue in mammalian forms of HAS. In mouse, a mutation to Arg results in complete activity loss^24^. In the absence of any mutational studies in SeHAS, to further elucidate its role, we conducted mutation studies of Lys-139. Details on the experimental setup and assay are described in *Methods*. Experimental results and pMBAD vector map used for the study are shown in Fig. 4A,B, respectively. We mutated Lys-139 to Arg (K139R) with the aim of conserving the charge at that position. Mutation did not lead to complete loss of activity, but did have an impact. Only 34% activity (HA production) was retained in comparison with control which reiterates a significant functional role of this residue (Fig. 4A). To further probe the charge-based interaction between this residue and substrate, we mutated positively charged Lys-139 to negatively charged Asp (K139D). Negative charge at the site leads to retention of 82% activity (HA production) in comparison with control. This analysis suggests a role for this residue in stabilizing polar groups of the substrate. The selection of substitutions further illustrate that both positive and negatively charged groups are accommodated at the site. Our studies show this residue directly influences synthase activity in SeHAS. Mutations at this site impair synthase function to considerable levels.

### SSE5 binds to polymer and influences HA production

The cavity above the UDP-substrate binding region formed by amphipathic helices is evaluated for binding of polymer unit. The polymer binding study is limited to the terminal disaccharide involved in glycosidic linkage formation. Docking studies were conducted with HA disaccharide unit comprising of a β 1–4 linked N-acetylglucosamine and D-Glucuronic acid in glycosyltransferase domain. Energetically favourable conformers were selected. Figure 3D shows a HA disaccharide unit docked near the binding pocket. SSE5, SSE8 and SSE9 are involved in binding (Fig. 3D). Residue Asp-260 from SSE8 is in H-bonding distance to terminal OH group of the polymer and is indicated by a dashed line in Fig. 3D. Arg-261 from SSE8 lies close to the polymer and could interact with the polar OH groups of the sugar. Residue Trp-299 from SSE9 forms CH-π interactions with the terminal sugar.

To the best of our knowledge, this is the first report hypothesizing the functional involvement of residues from SSE5. Key residues in SSE5 involved in binding to the polymer are Arg-205, Tyr-206, Phe-210 and Arg-214. Arg-205 is in close proximity to the docked disaccharide and involved in interactions with the negatively charged polar groups of sugar moiety. Tyr-206 or phenylalanine in SeHAS homologues is seen to stabilize the Arg-205 residue through cation-π interactions. Arg-214 or Lys in homologous sequences is in close proximity to the polymer and close to partially conserved Phe-210 with which it forms cation-π interactions. The consequence of mutation of the residues from SSE5 is not known. Here, we probe the consequence of mutating Tyr-206 and Arg-214 on function through site-directed mutagenesis. Our experimental results show that disruption of cation-π interactions by mutating the aromatic tyrosine to alanine (Y206A) results in 99% loss of HAS activity (HA production) (Fig. 4A). Loss of this interaction could have indirectly influenced polymer binding through adjacent Arg-205. Similarly, mutation of basic arginine residue at 214 to alanine (R214A) leads to complete loss of function (Fig. 4A). Interestingly, introduction of negative charge (R214E) leads to 93% function loss (Fig. 4A). These results iterate the need for a net positive charge at this site in order to facilitate polymer binding and translocation. These mutations indicate the interdependence of polymer binding/translocation and glycosyltransferase activities.

Further, the enzyme complexed with the two ligands (UDP-sugar and disaccharide) was energy minimized using AMBER force fields. Figure 3F shows the enzyme bound to UDP-N-acetylglucosamine and disaccharide formed by linked glucuronic acid and N-acetylglucosamine with glucuronic acid at the polymeric end. The details on the minimization protocol are described in *Methods*.

### Gln-248 (SSE7) is critical with a plausible role in conformation transition

Gln-248 is a conserved residue in SSE7, a long loop away from binding site. The equivalent loop in both processive and non-processive homologues is sequentially different and shows distinct conformations in substrate bound and unbound enzyme forms (Fig. 3E(ii))^29–31^ and Fig. 3E (iii) β 1–4 galactosyltransferase, PDB code: 2FYD and 2FY7)^32^. The loop in SeHAS is likely to facilitate positioning of SSE8 for catalysis. Residue Gln-248 lies away from the binding pocket; the exact role of this residue could not be predicted based on these *in-silico* studies. In-house mutation studies show this residue to be important. Replacement of the residue with Ala results in near complete loss of HA production (Fig. 4A).The role of this residue is further probed through coarse grained dynamic studies and is discussed in a later section.

### SSEs exhibit coordinated motion

**Figure 5:**
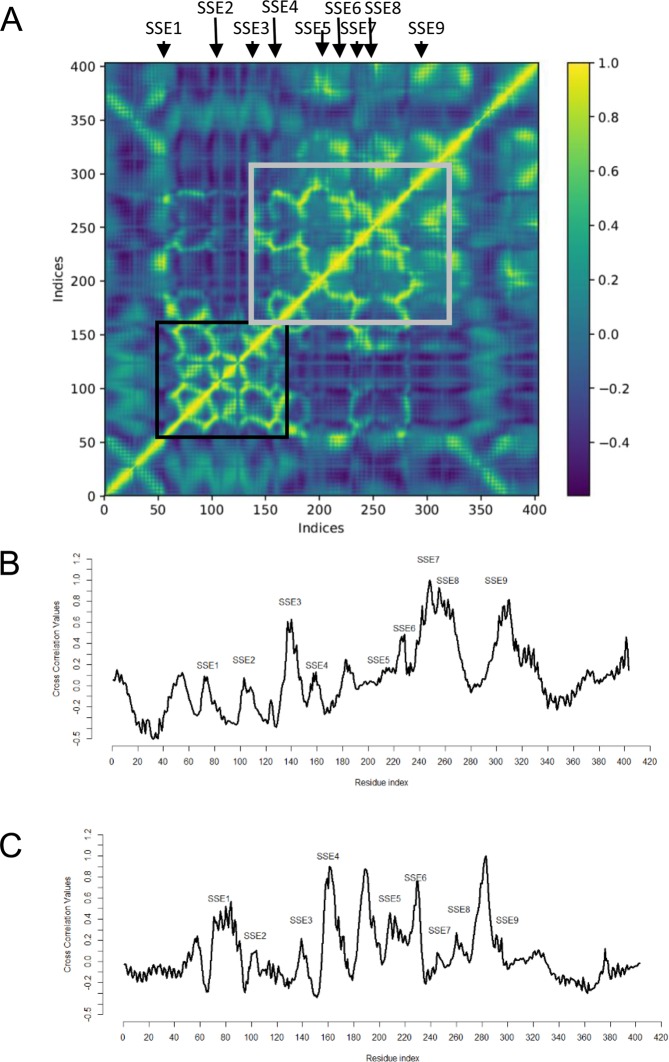
Assessment of global dynamics in SeHAS. (**A**) Average correlation coefficient values for SeHAS across residue pairs. Average Correlation coefficient values for Gln-248 (**B**) and Thr-283 (**C**) with other SeHAS residues.

The current paradigm is structure-encodes-dynamics-encodes-function^33–35^. It has increasingly being recognized that random fluctuations of atoms in their native state conceal coordinated motion that predispose the protein for functionally relevant changes in structure. These motions can be captured by low frequency modes in elastic network modeling. These modes are known to be insensitive to structural and energetic details. Hence we used Anisotropic Network Modeling (ANM), an ENM based approach to capture global image of dynamics encoded by 3-D structure of SeHAS. We used ProDy^36^ for ANM computations. Further details are elaborated in *Methods* section. Coordinated motion is represented as correlation coefficient values between nodes of the ANM network. The average correlation coefficient values were computed based on the first 50 modes. Figure 5A shows the correlation map for SeHAS. Positive correlation coefficient values are indicative of atoms moving in same direction. Cluster of spatially proximal residues with high correlation coefficient are considered to be structurally and functionally important. The residue pairs with large positive values correspond to sub-structural elements. Further, the correlation between residue pairs extends to regions across different SSE. SSE1-4 form a closely interacting subdomain (black rectangle). Similarly, SSE5-9 form a coordinated interacting subdomain (grey rectangle). The two subdomains are separated by a poorly correlated region. The intrinsic global motions illustrate functional coordination required with respect to substrate binding at one end, by SSE1-4 and polymer binding at the other end (SSE5-9).

We further explored the dynamics of SeHAS with respect to residue Gln-248. Fig. 5B shows the distribution of correlation coefficient values with respect to other residues in SeHAS. The Gln-248 show large positive values with SSE8, 9 and SSE3 which hold catalytic and substrate binding residues. A coordinated motion with these SSEs assessed through ANM further illustrates its role in functionally relevant conformation dynamics.

The ANM study is extended to assess the effect of mutation on the correlation coefficient values. For each of the mutant, a structural model is obtained using RaptorX^26^. The mutants show high structural overlap with an average RMSD of about 1 Å across all atoms with respect to the WT protein structure. For every mutant structure, correlation coefficient values with respect to mutant residue are computed and compared with the WT. The plots are shown in Fig. S2. Mutations at site 214, 248 and 283 exhibit substantial difference in values in SSE regions.

### Reducing end polymer elongation in SeHAS, a plausible three-step mechanism

**Figure 6:**
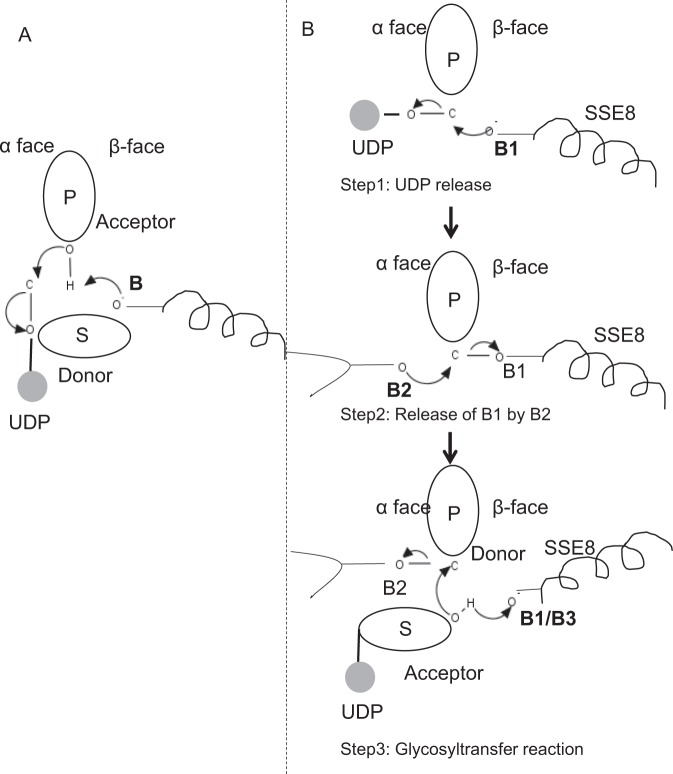
Proposed molecular mechanism in SeHAS. (**A**) Mechanism in a prototypic glycosyltransferase with inversion chemistry. (**B**) Proposed mechanism in Class I HAS. P: Polymeric sugar, S: Substrate sugar.UDP is indicated as a circle. B1, B2 and B3 are the catalytic bases participating in the reaction mechanism.

In prototypic members of glycosyltransferase family GT-2 such as cellulose synthase, the polymer biosynthesis occurs from the non-reducing end of growing polymer^25,31^. In such systems, UDP-substrates act as a donor transferring the sugar to the polymeric sugar which acts as a acceptor^24^. A β-linkage between the sugars is created from α-linked sugar-UDP precursors through a direct displacement S_N_2 substitution reaction. In this reaction, a deprotonation step by a catalytic base functionalizes the acceptor^24^. Nucleophilic attack by this acceptor sugar group onto the donor via a single oxocarbenium ion-like transition state results in glycosyl transfer reaction with the release of UDP from the donor sugar and net inversion of stereochemistry at anomeric carbon. This mechanism is illustrated in Fig. 6A. SeHAS differs from this prototype. It is known that HA elongates from the reducing end^12^. This implies (i) UDP is released from polymer end and not UDP-substrates during catalysis and (ii) Reversal of donor and acceptors with HA-UDP acting as donor and UDP-sugar substrate as acceptor. Based on the landscape of ligand-binding and obtained knowledge about active site architecture, we discuss the participation of ligands as donor and acceptor and propose a catalytic mechanism for glycosyl transfer in SeHAS (Refer Fig. 6B).

#### Glycosyltransferase reaction likely occurs in multiple steps

As the first step, we explored the possibility of a single step glycosyltransferase reaction in cellulose synthase-like manner. If the glycosyltransferase reaction occurs in one step, the enzyme at a given time would need to hold two UDP moieties, one attached to polymer and other attached to incoming substrate. In the alternate multi-step scenario, the UDP from polymer is released before catalytic reaction occurs. In this case, to create net inversion of stereochemistry, reaction would occur in a series of odd number of steps with S_N_2 mechanism. To evaluate which scenario is more likely, we conducted UDP binding studies through docking of UDP molecules in the glycosyltransferase domain. The assessment indicates a significant overlap of UDP and UDP-substrate binding sites.The result is presented in Supplementary Section. One exemplary energetically favourable conformation of UDP along with substrate and polymer binding regions are shown in Supplementary Fig. S3. Though the assessment is based on the static model, placement of two large molecules of similar chemical nature in close proximity with requirements of similar physiochemical environments is unlikely for this enzyme. We speculate that a passage lined with positively charged residues from SSE9 and metal ion could facilitate UDP release from the polymer end. Involvement of metal ion in glycosyltransferase for UDP release is also well documented^24^. Since the two UDP sites are not mutually exclusive, the second scenario of multi-step reaction appears more likely.

Once UDP is released from polymer, a nucleophilic attack from the OH group of substrate–UDP (acceptor) would create a glycosidic linkage. To create inversion, UDP release and subsequent nucleophilic attack must occur from two opposite faces of polymeric sugar. We propose the role of three base catalysts in overall reaction scheme which lie on α and β faces of the donor sugar at the polymer end. These steps are illustrated through Fig. 6B and described below.

#### Proposed three-steps glycosyltransferase reaction

Step1: UDP release: Asp-260 from SSE8 could act as a base (B1) and initiate the release of UDP molecule from polymer (donor) from α-face. This step would create a covalently bound glycosyl enzyme intermediate shielded on β face with the base B1. To create an inversion this face of the sugar needs to be free for nucleophilic attack by incoming sugar O-H group. Hence an intermediate step, with the nucleophilic attack from α face would release the β face ready for nucleophilic attack by acceptor sugar.

Step 2: Release of B1 by B2; Exploring the role of Thr-283: Base B2 on α face of donor sugar would carry out a nucleophilic attack on the anomeric carbon at the reaction center and release B1. Our structural studies show conserved Gln-295 and Thr-283 on this face. UDP release might result in conformational changes and bring one of these residues in proximity to donor sugar for catalysis. Mutation of Gln-295 results in up to 90% loss of activity but the role as a base is not known^20^. We assessed the possibility of a conserved Thr-283 residing in a loop to function as the second base. We mutated Thr to Ser containing a similar functional OH group and to Ala with small aliphatic side chain. Both, T283A and T283S mutants show ~95% loss of HA production (Fig. 4A). This residue exhibits positive correlation with regions SSE4 and SSE6 and a spatially proximal region between residue 186 and residue 190 (Fig. 5C). Residue 188, though not conserved, is in the vicinity of docked substrate (Fig. 3). Thr-283 does not show high positive correlation with SSE8 holding the catalytic base. It is possible that the residue acts as a supporting base residue during catalytic reaction with its motion coordinated with spatially proximal substrate binding region independent of Asp-260 base in SSE8. Further experiments would be required to substantiate its role as a base. Overall, the study highlights yet another functionally critical residue, not known previously.

Step 3: Glycosyl transfer reaction: Once B1 is released, β face of the anomeric carbon of the polymeric sugar is unblocked for the reaction. The deprotonation of substrate-UDP on the same face by a third base would create a nucleophilic group. This step could be initiated by B1 or another base B3. Asp-259, next to B1 is a highly conserved residue in proximity to substrate-UDP and could act as B3. However, B1/B3 is closer to the polymeric sugar (donor) as discussed in Step1. To initiate deprotonation, B1 has to be in proximity to acceptor sugar. It could be achieved through conformation changes at SSE8 assisted by SSE7. The equivalent helix in cellulose synthase holds catalytic base, and undergoes coordinated conformational transitions for glycosyltransferase reaction and translocation. A nucleophilic attack of the acceptor sugar on the anomeric carbon of the donor sugar would create a glycosidic linkage and release of base B2.

## Concluding Remarks

Due to the absence of SeHAS 3D structure, the understanding on Class I HAS functioning is limited. The gap in knowledge poses challenge in rational engineering of HAS to modulate HA properties. The present study provides a dictionary of substructural elements of functional importance that could act as framework for rational engineering of HAS class I family of enzymes. The 3-D structural model developed in this study sheds light on membrane organization in SeHAS. Docking studies suggest overlap of the binding sites of UDP-N-acetylglucosamine and UDP-D glucuronic acid. The dual specificities could therefore be guided by conformational changes to create preference for one substrate over the other. These evidences favour the overall pendulum hypothesis model with two binding sites, one for acceptor and one for donor with alternating specificities^12^. Structural and mutation studies establish Lys-139 from SSE3 to be dispensable for SeHAS functioning with a role in binding to substrate. *In-silico* and mutation studies provide evidence for the influence of polymer binding region, located in SSE5, a membrane-cytosolic interfacial region, distant from the UDP-substrate binding site, on HAS function. Based on insights derived on active site architecture, ligand coordinating regions and functional requirements for HA biosynthesis process, we propose a three-step mechanism to extend polymer from reducing end. The present analyses suggest that the release of UDP from polymeric end would be required for glycosyltransferase reaction. Coarse grained dynamic assessment of SeHAS reveals collective motion of SSE.

In future, molecular dynamics simulations for substrate selectivity and translocation of polymer would be insightful for investigating intricate conformational dynamics essential for catalysis. A complete molecular level understanding of biopolymer synthesis will enable structure-guided enzyme engineering to modulate desired biological properties such as substrate composition, molecular weight and other parameters of interest. The findings could be extended to other processive glycosyltransferases with little functional information. We believe this work would add a significant step and prompt further research to understand molecular mechanism of HA synthesis as well as for rational design of HAS and related enzymes.

## Methods

### Structure modelling and assessment

Structure model of SeHAS sequence is generated using RaptorX^26^ webserver. The software is optimized for targets with no close templates. It integrates several features such as sequence similarity, secondary structure, disorder, domain information, profile entropy from target sequence and template structures to assess, optimize and score threading alignments. The software constructs 3-D models for the top ranked templates. Each predicted model is associated with measures such as P-value, alignment score and Global Distance Test score for quality assessment. We evaluated stereochemical quality of the predicted model using RAMPAGE server^37^. For structure comparison and superimposition, we used Mustang^38^. We used open source version of PyMol for 3-D structure visualization and structure analysis^39^.

### Homologue detection and sequence comparison

Homologues of SeHAS sequence are obtained using BLAST^40^ search against UniProt database^41^ using SeHAS as query sequence. Sequences similar to SeHAS are identified using strict similarity search measures and manual scrutiny. The obtained hits were filtered based on (i) annotation, (ii) query coverage (>=70%), (iii) sequence identity (>25%) and (iv)e-value (<0.001). Only full length sequences with predicted transmembrane region are considered for analysis. We used ClustalW tool to generate sequence alignment of SeHAS and its homologues^42^. We computed conservation score for every residue position in SeHAS with respect to its homologues. It is calculated as percentage of identical residues for the number of aligned residues at that site averaged over a window of 5 residues. Links to the softwares and algorithms are provided as Supplementary Table 1.

### Protein-Ligand docking analysis

AutoDock 4.2 tool^43^ is used to conduct docking simulations of ligands onto the protein structure. In all simulations, protein is kept rigid and ligand flexible with movement around single rotatable bonds in order to capture energetically favourable binding pose of the ligands. We used standard AutoDock protocol using genetic algorithm for simulations. For UDP-substrates, docking area is defined by a box of 48 × 40 × 40 Å with 0.375 Å spacing in the glycosyltransferase domain. Lamarckian genetic algorithm with 150 randomly placed entities, 27000 generations, 25000000 energy evaluations, a mutation rate of 0.02, a cross over rate of 0.8 with a total of over 50 runs per compound. The docking simulations were conducted without the disordered C-terminal loop. A cut off of −5 kcal/mol is chosen to shortlist ligand poses with energetically favourable binding. The shortlisted binding pose for the two UDP-substrates are manually scrutinized to select biologically relevant conformers. HA disaccharide coordinates comprising of β-linked N-acetylglucosamine and D-glucuronic acid is used to assess polymer binding region. The docking area was defined by a grid of 40 × 40 × 54 Å with 0.375 Å spacing and its centre defined as intersection of axes perpendicular to three amphipathic helices. Energetically favourable linear conformers above the UDP-substrate binding region are selected as relevant conformers for further study.

### ANM analysis

The ANM model for SeHAS was generated using ProDy^36^ with default parameters. The Cα atoms of the protein were represented as nodes of the network connected by springs with a distance cut-off of 15 Å. The correlation is calculated as normalized inner product of displacement vector of two atoms averaged over all modes and time^36^. The values range between −1 and 1.

### Energy minimization

The enzyme structure docked with UDP-substrate and disaccharide was energy minimized using open source binaries of Ambertools suite version 19^44^. Energy minimization of the complex was performed using Sander program with implicit solvent. The simulations were run for a maximum of 5000 time steps. Force fields ff14SB and gaff were used for proteins and ligands, respectively. Tleap program was to create topology and parameter files. Intermediate files required for tleap program were prepared using antechamber.

### Bacterial strain and growth conditions

*Escherichia coli* TOP10 cells were grown on Luria Bertani-Miller (LB) agar and LB liquid medium. Genetically modified *E*. *coli* TOP10 cells were grown on LB agar and liquid medium supplemented with 100 µg/ml of amplicillin (LB + amp). Chemically competent cells of *E*. *coli* TOP10 were prepared using calcium chloride as mentioned in Sambrook^45^. Supplementary Table 1 lists the source for various experimental model systems and reagents used in this study.

### Construction of clones for mutational studies

Codon optimized hyaluronan synthase (*has*A), hyaluronan synthase mutants (*has*A*) and UDP-glucose 6-dehydrogenase (*has*B) from *Streptococcus equi subsp*. *zooepidemicus* (GenBank: AF414053.1) were synthesized as a gene set (SeHAS-AB/SeHAS-A*B) and purchased from Invitrogen GeneArt Gene Synthesis (ThermoFisher Scientific, USA) for expression in *Escherchia coli*. List of hyaluronan synthase mutants (*has*A*) are as follows: K139D, K139R, Y206A, R214A, R214E, Q248A, T283A and T283S. Gene sets (*sehas*AB/*sehas*A*B) were PCR amplified with forward primer (*NcoI*) 5′-GTGGT*CCATGG*GTCGTACCCTGAAAAATCTGA-3′ and reverse primer (*HindIII*) 5′-GGATC*AAGCTT*TCATTAATCGCGACCA-3′ using Phusion® High-Fidelity DNA Polymerase (New England BioLabs® Inc., USA). Amplicons were purified with NucleoSpin® Gel and PCR Clean-up kit (MACHEREY-NAGEL GmbH & Co. KG, Germany). Expression vector pMBAD was kindly provided by Prof. Yu, Huimin, Department of Chemical Engineering, Tsinghua University, P. R. China^46^. pMBAD and the amplicons were digested with NcoI and HindIII restriction enzymes as per manufacturer’s protocol (New England BioLabs® Inc., USA). Restricted digest products were purified as mentioned above and ligated using T4 DNA Ligase (New England BioLabs® Inc., USA) as per manufacturer’s protocol to obtain pMBAD-*sehas*AB or pMBAD-*sehas*A*B constructs. Recombinant plasmids were transformed into chemically competent expression host *E*. *coli* TOP10 cells by heat shock method^45^.

### Expression study and analyses

Following procedure applies to both wild type TOP10-pMBAD-SeHAS-AB and TOP10-pMBAD SeHAS-A*B mutants. Single colony was inoculated in 10 mL of LB + amp liquid medium and grown overnight at 37 °C and 200 rpm. Overnight culture was inoculated in 50 mL LB + amp (0.05% inoculum) supplemented with 20 mM MgCl_2_ and grown at 37 °C and 250 rpm. The culture was induced at 0.8 OD_600_ with 0.1 g/L of *L*-Arabinose and grown at 30 °C and 180 rpm after induction. After 5 hours, 2.5 g/L of K_2_HPO_4_, 1 g/L of sorbitol and 10 g/L of glucose were added separately to further improve HA production. Biomass (OD@600 nm) was measured at regular intervals using BioPhotometer Plus (Eppendorf, Germany) whereas HA concentration was measured after 24 hours. Fermentation broth was treated with equal volume of 0.1% SDS and mixed at room temperature for 20 minutes to remove capsular HA. After centrifugation, supernatant was treated with 4 volumes of absolute ethanol and stored at 4 °C overnight. Precipitate was collected after centrifugation and re-suspended in 0.1 M NaCl for estimation of HA concentration by modified carbazole method^47^.

## Supplementary information

Supplementary file

## Data Availability

The data generated or analysed during the current study are available from the corresponding author on reasonable request. Further details on data source are provided in Supplementary Information.
