# Supplementary material for: Biosynthesis of Hyaluronic acid polymer: Dissecting the role of sub structural elements of hyaluronan synthase
Source: Sci Rep. 2019 Aug 29;9:12510. doi: 10.1038/s41598-019-48878-8 (PMC6715743; doi:10.1038/s41598-019-48878-8)
Supplement: Supplementary file 1 — Supplementary file [file 41598_2019_48878_MOESM1_ESM.docx]

**Biosynthesis of Hyaluronic acid polymer: Dissecting role of sub structural elements of hyaluronan synthase**

**Authors**

GarimaAgarwal^a^,*, Krishnan KV^b^, Shashi BalaPrasad^b^, AnirbanBhaduri^a^, GuhanJayaraman^b^,*

Supplementary Table 1: List of resources and the respective source

| **REAGENT or RESOURCE** | **SOURCE** | **IDENTIFIER** |
| --- | --- | --- |
| *Bacterial and Virus Strains* | | |
| *Escherichia coli* TOP 10 | Invitrogen | C404010 |
| *Chemicals, Peptides, and Recombinant Proteins* | | |
| Luria Bertani Broth, Miller | HIMEDIA | M1245 |
| Luria Bertani Agar Miller | HIMEDIA | M1151 |
| Ampicillin Sodium Salt (Amp-Na) | SISCO RESEARCH LABORATORIES PVT. LTD. | 61314; CAS: 69-52-3 |
| Calcium chloride (CaCl_2_.2H_2_O) | HIMEDIA | RM534; CAS: 10035-04-8 |
| L-Arabinose | HIMEDIA | GRM037; CAS: 5328-37-0 |
| D-Glucose | FISHER SCIENTIFIC | 15405; CAS:50-99-7 |
| di-Potassium hydrogen phosphate anhydrous (K_2_HPO_4_) | MERCK | 1.93230.0521 |
| D-Sorbitol | HIMEDIA | GRM109; CAS: 50-70-4 |
| Magnesium chloride (MgCl_2_.6H_2_O) | SISCO RESEARCH LABORATORIES PVT. LTD. | 1349117; CAS: 7791-18-6 |
| Sodium Lauryl Sulphate | SISCO RESEARCH LABORATORIES PVT. LTD. | 54468; CAS: 151-21-3 |
| Carbazole | FLUKA | GA20524 |
| Ethanol | HAYMAN | F204325 |
| Sodium chloride (NaCl) | MERCK | 1.93206.0521 |
| Sodium nitrate (NaNO_3_) | MERCK | 1.93631.0521 |
| Hyaluronan synthase (*has*A) | Invitrogen GeneArt Gene Synthesis, ThermoFisher Scientific (GenBank: FM204884) | Accession: YP_002743732 |
| UDP-glucose 6-dehydrogenase (*has*B) | Invitrogen GeneArt Gene Synthesis, ThermoFisher Scientific (GenBank: FM204884) | Accession: YP_002743733 |
| Hyaluronan synthase mutants (*has*A*) | Invitrogen GeneArt Gene Synthesis, ThermoFisher Scientific | This study |
| Critical Commercial Assays |  |  |
| NucleoSpin® Gel and PCR Clean-up kit | MACHEREY-NAGEL GmbH & Co | 740609.50 |
| NucleoSpin® Plasmid | MACHEREY-NAGEL GmbH & Co | 740588.50 |
| EmeraldAmp® MAX PCR Master Mix | TAKARA BIO INC., | RR310Q |
| *Experimental Models: Organisms/Strains* | | |
| *Escherichia coli* TOP 10 | Invitrogen | C404010 |
| *Oligonucleotides* | | |
| FP- GTGGTCCATGGGTCGTACCCTGAAAAATCTGA | This study | N/A |
| RP- GGATCAAGCTTTCATTAATCGCGACCA | This study | N/A |
| Recombinant DNA | | |
| Plasmid: pMBAD | Prof. Yu, Huimin, Department of Chemical Engineering, Tsinghua University, P. R. China | N/A |
| Software and Algorithms | | |
| BLAST | (Altschul et al., 1997) | https://blast.ncbi.nlm.nih.gov/Blast.cgi |
| ClustalW | (Sievers et al., 2011) | https://www.ebi.ac.uk/Tools/msa/clustalw2/ |
| RaptorX | (Källberg et al., 2012) | http://raptorx.uchicago.edu/StructurePrediction/predict/ |
| RAMPAGE | (Lovell et al., 2003) | http://mordred.bioc.cam.ac.uk/~rapper/rampage.php |
| Autodock | (Morris and Huey, 2009) | http://autodock.scripps.edu/downloads/autodock-registration/autodock-4-2-download-page/ |
| MUSTANG | (Konagurthu et al., 2006) | http://lcb.infotech.monash.edu.au/mustang/ |
| PyMol | (DeLano, 2002) | https://github.com/schrodinger/pymol-open-source |
| Other | | |
| Phusion® High-Fidelity DNA Polymerase | NEW ENGLAND BIOLABS® INC., | M0530S |
| T4 DNA Ligase | NEW ENGLAND BIOLABS® INC., | M0202S |
| NcoI | NEW ENGLAND BIOLABS® INC., | R3193S |
| HindIII | NEW ENGLAND BIOLABS® INC., | R3104S |

***Identification of sub-structural elements (SSE)***

**Figure S1:** Multiple sequence alignment of Class I HAS enzyme sequences.SSE elements and potential catalytic base indicated in a rectangle.

**
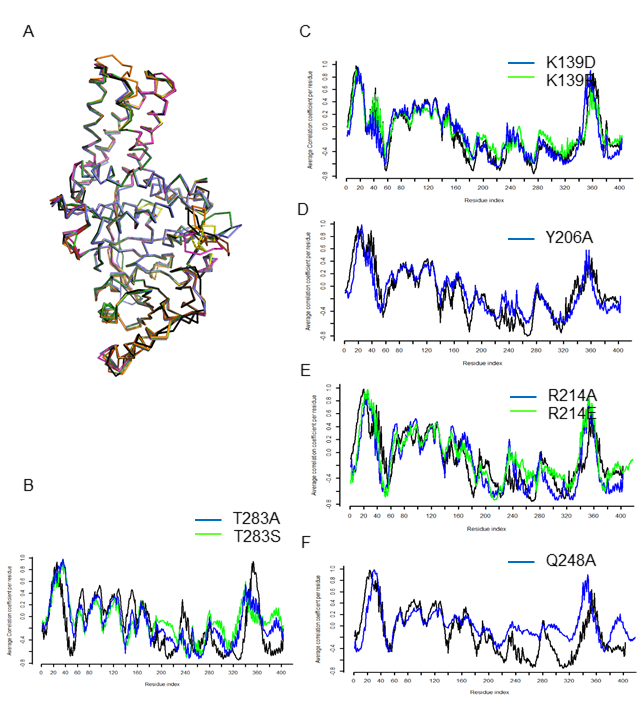
**

**Figure S2: Assessment of mutant structures A) Superimposition of mutant structures over the WT structure (shown in black) B)-F) Distribution of average correlation coefficient values per residue for WT and mutants. WT is shown in black.**

***Molecular mechanism for polymer elongation***


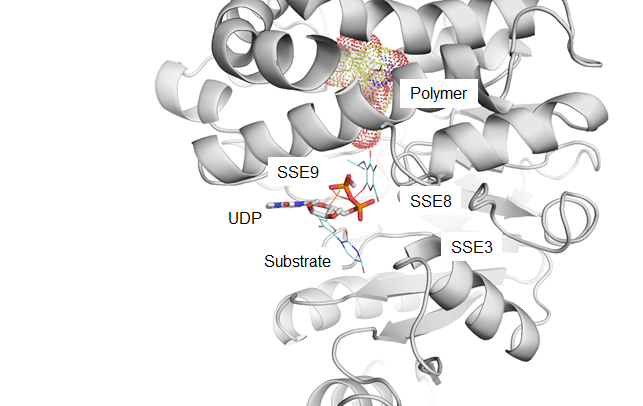


**Figure S3:** UDP binding and its overlap with UDP-substrate binding site. The UDP-substrate binding region is highlighted in green dots and the polymeric region in blue dots. UDP is shown in stick representation.
